# Parental bonding style and illness severity in a longitudinal study of individuals with first episode psychosis

**DOI:** 10.1007/s00737-023-01351-y

**Published:** 2023-07-28

**Authors:** Rebecca Pollard, Helen L Fisher, Paul Fearon, Kevin Morgan, Julia Lappin, Gerard Hutchinson, Gillian A. Doody, Peter B. Jones, Robin M. Murray, Craig Morgan, Paola Dazzan

**Affiliations:** 1 Department of Psychological Medicine, Institute of Psychiatry, Psychology & Neuroscience, King’s College London, 16 De Crespigny Park, London, SE5 8AF UK; 2 Social, Genetic & Developmental Psychiatry Centre, Institute of Psychiatry, Psychology & Neuroscience, King’s College London, London, UK; 3 ESRC Centre for Society and Mental Health, King’s College London, London, UK; 4 Discipline of Psychiatry, School of Medicine, Trinity College, Dublin, Ireland; 5 Department of Psychology, University of Westminster, London, UK; 6 School of Psychiatry, Faculty of Medicine, UNSW, Sydney, Australia; 7 Department of Psychiatry, Faculty of Medical Sciences, The University of the West Indies at Saint Augustine, Saint Augustine, Tunapuna–Piarco, Trinidad and Tobago; 8 Department of Psychiatry, University of Nottingham, Nottingham, UK; 9 Department of Psychiatry, University of Cambridge, Herchel Smith Building, Cambridge, CB2 0SZ England, UK; 10 Department of Psychosis Studies, Institute of Psychiatry, Psychology & Neuroscience, King’s College London, London, UK; 11 Department of Health Service & Population Research, Institute of Psychiatry, Psychology & Neuroscience, King’s College London, London, UK

**Keywords:** Schizophrenia, First episode psychosis, Parental bonding

## Abstract

A parenting style with high amounts of control combined with low caring or nurturing behaviour has been reported in association with mental disorders including schizophrenia. However, the association of parenting style with illness severity in individuals with schizophrenia has never been evaluated retrospectively or over a longitudinal time course. In a subset (*n* = 84) of the participants included in the AESOP (Aetiology and Ethnicity in Schizophrenia and Other Psychoses)-10 study, we evaluated participants’ perceptions of their own parents’ bonding style at the time of their first episode of psychosis using the parental bonding instrument (PBI). We then examined the association between different bonding styles, illness course and severity, and global functioning over a 10-year follow-up. Participants who perceived that their fathers had a more caring and less controlling parenting style showed better functioning at follow-up. However, in contrast to previous research, participants who reported having been subject to uncaring and controlling parenting styles were not found to have a notably worse course of illness or symptom severity over the follow-up period. These results indicate that more optimal parental bonding styles may be associated with better overall functioning in individuals with psychosis but not with other measures of illness outcome.

## Introduction

Studies that have operationalised parental bonding show there are two main concepts that contribute to the parental bonding relationship: warmth and affection over coldness and neglect, and excessive control, defined by strict rules and intrusiveness (Roe and Siegleman, 1963). An ‘optimal’ parental bonding style as defined by Bowlby (1977) consists of adequate care and nurture, but involves the promotion of independence.

Sub-optimal, affectionless-control, styles of parental bonding have been found to be more prevalent in individuals diagnosed schizophrenia than in unaffected individuals (Winther Helgeland and Torgersen 1997). In affected individuals, perceived low-care, high-control parenting styles are associated with a non-remitting course of illness (Parker et al. 1982), earlier onset of illness (Baker et al. 1984) or more days of inpatient admission (Parker et al. 1982; Warner and Atkinson 1988).

However, previous studies investigating the impact of bonding styles on illness outcomes have used cross-sectional designs or short follow-up periods of 12 months or less (Baker et al. 1984; Parker and Mater 1986; Warner and Atkinson 1988). There is a lack of evidence of the association between premorbid parental styles and outcomes over longer follow-up periods in individuals with psychosis.

Uniquely, this study investigates the relationship between parental bonding evaluated at the first presentation for a psychotic disorder and outcomes in the subsequent 10 years of illness. Based on previous literature, we hypothesised that participants with perceptions of ‘affectionless control’ parenting styles would be more likely to experience a non-remitting course of illness, more severe symptoms and poorer global functioning over 10 years.

## Methods

The sample represents a subset of the AESOP-10 sample (Morgan et al. 2006). AESOP-10 is a 10-year follow-up of 557 individuals, aged 16–64 years who were recruited when presenting to services with a first episode of psychosis (ICD-10 codes F20–F29 and F30–F33) across sites in South-East London and Nottingham (Morgan et al. 2014). Exclusion criteria included diagnoses of transient episodes of psychosis, organic causes of psychosis, head injury or lack of English comprehension. Full details of participant recruitment and follow-up procedures are detailed in previous publications (Morgan et al. 2006; Morgan et al. 2014).

### Parental bonding instrument (PBI)

The PBI (Parker et al. 1979) was completed at baseline by 84 participants at the Nottingham site. The PBI consists of 2 subscales (control and care), which are administered to participants once for each parent. Raw scores were calculated for each subscale. According to the protocol of Warner and Atkinson (1988), we calculated a continuous ‘risk score’ with lower scores indicating that parents were perceived to have shown high protection and low care behaviours thus demonstrating a sub-optimal parental style.

### Clinical and functional outcome over the first 10 years of illness

Illness course over 10 years was assessed retrospectively. Information was collated from clinical records and, where possible, from follow-up interviews with participants and treating clinicians using an extended version of the World Health Organization (WHO) Life Chart (Sartorius et al. 1996). Participants who were followed up did not differ in symptom severity at baseline, according to ICG scores, from those who were not (*z* = −1.62, *p* = 0.11). Nottingham participants showed lower symptom severity at baseline compared to London participants (*z* = −2.36, *p* < 0.05).

Participants were classified into having a ‘non-remitting’ or ‘remitting’ course of illness and symptom severity during each episode was assessed using Rating Scale 2 of the SCAN (Wing et al. 1990) which scores usual symptom severity during a psychotic episode on a scale from 0 to 3, as either absent, mild, moderate or severe. The average symptom severity across all episodes over the follow-up period is used here.

Level of functioning at the 10-year follow-up was assessed with the Global Assessment of Functioning (GAF) scale with symptom (GAF-s) and disability (GAF-d) subscales (Hall 1995).

Illness severity at baseline was calculated using a sum of the dimension scores from the Item Group Checklist (ICG) of the SCAN to compare between the full AESOP sample and those included in this analysis.

### Statistical analyses

Differences in PBI risk scores between participants with different illness courses and severity were estimated using ANOVA or independent *t*-test. Regression analyses were performed to evaluate the relationship between parental bonding and contribution of multiple covariates (age, sex, baseline diagnosis) on global functioning scores.

## Results

Socio-demographic and clinical characteristics of the sample are presented in Table 1. Most patients identified as White British (88%), and most (57%) had a diagnosis of non-affective psychosis. The mean paternal risk score was 12.6 (SD = 14) and the mean maternal risk score was 13.7 (SD = 14) (Table 2). PBI scores stratified by outcome groups are presented in Table 2. The mean GAF-s score was 67.09 (SD = 18.1), and mean GAD-d score was 65.89 (18.6). No differences were found in PBI scores between demographic groups.

**Table 1:** Demographic characteristics of the sample (*n* = 84)

	Total		Illness course				Usual symptom severity					
			Remitting illness course		Non-remitting illness course		Mild		Moderate		Severe	
	*n*	%	*n*	%	*n*	%	*n*	%	*n*	%	*n*	%
Male/female	38/35	52/48	10/17	37/63	19/17	53/47	7/11	39/61	27/21	56/43	1/3	25/75
White/other	64/9	88/12	26/1	96/4	30/6	83/17	2/16	11/89	5/43	10/90	1/3	25/75
Affective/non-affective psychosis	36/48	42/57	5/22	19/81	10/26	28/72	7/14	33/66	34/20	63/37	5/1	83/17
	Mean	SD	Mean	SD	Mean	SD	Mean	SD	Mean	SD	Mean	SD
Age	32.5	13	37.1	12.8	30.8	12.3	38	12	31	13	32	17
IQ	98.9	13.4	102.6	12.4	96.2	13.8	102	3	98	13	113	n/a

Values are displayed for the whole sample and for participants with a remitting and non-remitting course of illness. There were no significant differences between groups

**Table 2:** Parental bonding instrument (PBI) scores for the whole sample and for participants in the two main illness course groups

	Total		Remitting illness course		Non-remitting illness course		Severe usual symptoms		Moderate usual symptoms		Mild usual symptoms	
	Mean	SD	Mean	SD	Mean	SD	Mean	SD	Mean	SD	Mean	SD
PBI father protection score	10.7	7.8	10.7	8.2	11.7	7.9	11	8	11	8	10	6
PBI father care score	23.3	8.8	24.3	8.2	22.7	8.7	25	9	23	9	25	6
PBI father risk score	12.6	14.2	13.6	13.7	10.9	14.2	14	14	12	15	15	12
PBI mother protection score	11.7	7.8	13	8.1	10.9	7.4	13	8	11	8	12	4
PBI mother care score	25.3	9.9	25.2	10.6	24.2	8.9	24	12	25	9	28	6
PBI mother risk score	13.6	14.4	12.2	14.7	13.4	13.0	11	17	14	14	16	9

Parental bonding scores are presented as raw scores for each parent on both the care and protection subscales. Parental risk scores are calculated as a composite of these subscales (care score minus protection score) with higher scores indicating a more optimal parental bonding style. Values are displayed for the whole sample and for participants with a remitting and non-remitting course of illness. None of these values showed a significant difference between remitting and non-remitting illness groups

### PBI risk scores and clinical and functional outcomes

No differences were seen in paternal or maternal PBI risk scores between participants with a remitting or non-remitting course of illness or between participants with different illness severity (see Table 2).

A multiple regression analysis was carried out to investigate the association between PBI scores and global functioning. Only paternal PBI risk scores were included in the analysis as these were significantly correlated with GAF scores (GAF-s: *r* = 0.43, *p* < 0.05, GAF-d: *r* = 0.29, *p* < 0.05). Age, gender and baseline diagnosis were included as covariates. Symptom severity was not included as a covariate as it was not associated with either GAF or PBI scores. Paternal PBI risk scores (*β* = 0.37, 95% CI 0.13–0.82, *p* < 0.05) were a significant predictor of GAF-s scores at follow-up (*R*^2^ = 0.34).

## Discussion

Here we have evaluated, for the first time, the relationship between perceived parental bonding at the time of the first episode of psychosis and illness course and level of functioning over the first 10 years of illness. Our data show that the perception of sub-optimal paternal parenting at illness onset is associated with lower level of functioning 10 years later, but not with other clinical outcomes.

A perceived high care and low protection style of parenting by fathers as indicated by a lower paternal PBI risk score was associated with higher GAF scores at follow-up. Our results suggest that parental bonding styles may be associated with broad psychosocial functioning following a first episode of psychosis. This result indirectly supports findings from previous cross-sectional studies of individuals with a diagnosis of schizophrenia showing that this optimal parenting style is associated with a milder course of illness (Parker et al. 1982; Warner and Atkinson 1988). According to Bowlby’s theory of attachment (Giddens and Bowlby 1970) an affectionless control style in parenting will negatively impact a child’s psychosocial development due to a deprivation of adequate care and nurture and a failure to promote independence, which may be associated with emotional issues in adulthood. This might explain the results seen here.

We did not find sub-optimal parental bonding style to be associated with specific measures of illness severity in individuals experiencing a first episode of psychosis. This result is in contrast with previous findings that individuals with a diagnosis of schizophrenia who report unfavourable parenting styles are more likely to experience a more severe course of illness (Parker et al. 1982; Warner and Atkinson 1988). It is possible that the relatively small size of our sample may have prevented any potential association reaching statistical significance. Furthermore, our sample included participants with a first episode of both affective and non-affective psychoses, which differs from samples included in the studies mentioned above, many of whom were experiencing schizophrenia with a chronic course rather than their first episode. This could explain why our results are inconsistent with previous findings.

### Limitations

This study has some methodological limitations to be considered. We have a relatively smaller sample size which reduced the statistical power of our study. We included participants from just one study site, which had a lower average severity of illness at baseline. This might bias our results and restrict the generalisability of the findings.

While this study has its limitations, it gives a unique opportunity to consider the role of the perception of parental bonding early in the development of a psychotic illness and its impact on patient outcomes over an extended period.

## Conclusion

To our knowledge, this is the first study evaluating the impact of perceived parental bonding styles on functional and illness outcomes in individuals with psychosis after 10 years of illness. We found an association between ‘optimal’ paternal bonding styles and better long-term functioning in this sample. The observational nature of these data prevents any conclusion on a causal relationship between parental bonding styles and psychosis outcomes. Still, the results provide a basis for future studies on the role of parental bonding styles in individuals living with psychosis, with a view to improve familial support for these individuals and therefore improve functional and clinical outcomes.
